# SUMOylation and calcium control syntaxin-1A and secretagogin sequestration by tomosyn to regulate insulin exocytosis in human ß cells

**DOI:** 10.1038/s41598-017-00344-z

**Published:** 2017-03-21

**Authors:** Mourad Ferdaoussi, Jianyang Fu, Xiaoqing Dai, Jocelyn E. Manning Fox, Kunimasa Suzuki, Nancy Smith, Gregory Plummer, Patrick E. MacDonald

**Affiliations:** Alberta Diabetes Institute and Department of Pharmacology, University of Alberta, Edmonton, Alberta T6G 2R3 Canada

## Abstract

Insulin secretion from pancreatic ß cells is a multistep process that requires the coordination of exocytotic proteins that integrate diverse signals. These include signals derived from metabolic control of post-translational SUMOylation and depolarization-induced rises in intracellular Ca^2+^. Here we show that tomosyn, which suppresses insulin exocytosis by binding syntaxin1A, does so in a manner which requires its SUMOylation. Glucose-dependent de-SUMOylation of tomosyn1 at K298 releases syntaxin1A and controls the amplification of exocytosis in concert with a recently-identified tomosyn1-interacting partner; the Ca^2+^-binding protein secretagogin, which dissociates from tomosyn1 in response to Ca^2+^-raising stimuli and is required for insulin granule trafficking and exocytosis downstream of Ca^2+^ influx. Together our results suggest that tomosyn acts as a key signaling hub in insulin secretion by integrating signals mediated by metabolism-dependent de-SUMOylation and electrically-induced entry of Ca^2+^ to regulate the availability of exocytotic proteins required for the amplification of insulin secretion.

## Introduction

Insulin secretion from pancreatic ß cells occurs when the plasma glucose levels rise. Glucose is metabolized to increase the ATP: ADP ratio which subsequently increases the intracellular Ca^2+^ concentration ([Ca^2+^]_i_) to trigger the fusion of insulin granules with the plasma membrane^1^. In addition, glucose metabolism generates secondary coupling factors that amplify the secretion process^2^. We recently showed that the SUMOylation pathway acts as a downstream effector of secondary coupling factors, and works together with increased [Ca^2+^]_i_ to promote insulin exocytosis^3^. SUMOylation is the covalent conjugation of small ubiquitin-like modifier (SUMO) peptides to target proteins. The glucose-dependent activation of the SUMO protease SENP1 (Sentrin-specific protease 1) and subsequent de-SUMOylation of one or more exocytosis-regulating proteins augments the exocytotic response to Ca^2+^ entry^3^. Although much evidence suggests that the metabolic amplification of insulin secretion occurs at a distal site^4^, the effector molecules involved and their relationship to the Ca^2+^-sensing machinery is not completely understood.

A complex of soluble-*N*-ethylmaleimide-sensitive factor attachment protein receptors (SNAREs) tightly regulates the insulin secretion process. Two plasma membrane SNARE proteins, SNAP25 and syntaxin1A, and an insulin granule-associated SNARE protein VAMP2 compose the minimal SNARE complex in ß cells^1, 5^. Several auxiliary proteins add an additional level of control to SNARE complex assembly and insulin release^5^. Tomosyn was originally identified as a syntaxin1A-binding protein^6^, and shown to negatively regulate insulin secretion downstream of secretory granule docking^7, 8^ in a Ca^2+^-dependent manner^9^. In mammals, two genes that encode for tomosyn1 and tomosyn2 drive the expression of multiple isoforms^10, 11^. These may be recruited to a plasma membrane rich in syntaxin clusters, leading to the inhibition of syntaxin1A-SNAP25 binding^12^.

In response to glucose stimulation, tomosyn is post-translationally modified by phosphorylation and ubiquitylation^13^. Additionally, in neuronal cell lines the post-translational modification of tomosyn by SUMOylation adds a further level of regulation^14, 15^. Indeed, emerging evidence suggests multiple targets involved in exocytosis are subject to post-translational SUMOylation. This includes synaptotagmin VII^4^, syntaxin 1A^16^, Rim1α^17^, the Kv2.1 ion channel^18^, and synapsin 1A^19^. However, the SUMOylation of tomosyn and its role in the regulation of ß cell function have not been investigated.

Herein, we find that the SUMOylation of tomosyn in insulin-secreting cells is regulated by glucose. The SUMOylation of tomosyn1A is required for binding with syntaxin1A and suppression of exocytosis. Furthermore, we identify the Ca^2+^-binding protein secretagogin as a tomosyn-interacting protein in ß cells that is also required for insulin exocytosis, thus suggesting tomosyn as a key signaling hub controlling inputs from both metabolic coupling factors and [Ca^2+^]_i_ to regulate insulin secretion.

## Results

### SUMOylation controls tomosyn function in ß cells

**Figure 1:**
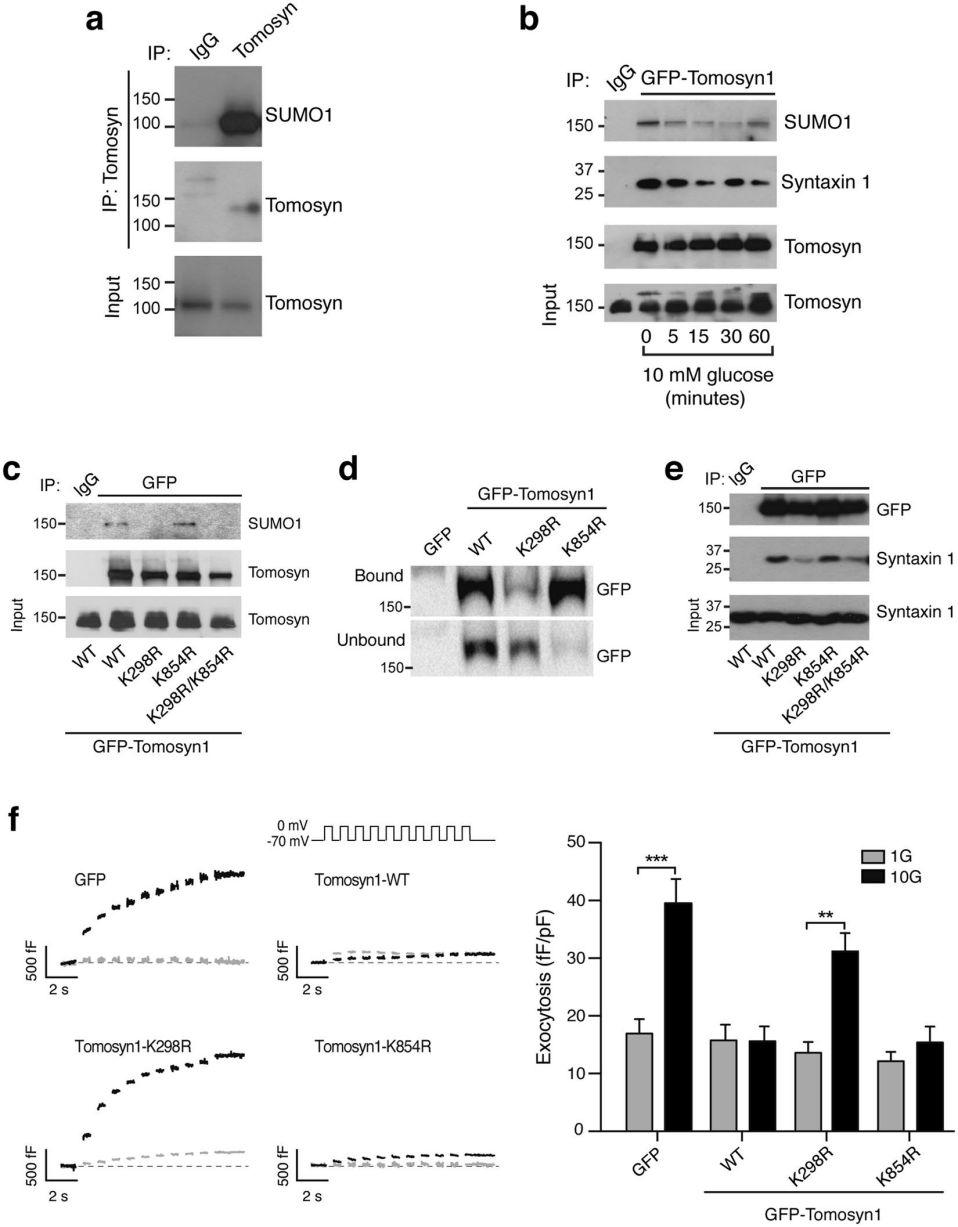
SUMOylation is required for tomosyn function in ß cells. (**a**) Pull-down of native tomosyn demonstrates the co-immunoprecipitation of SUMO1 in INS 832/13 cells (representative of n = 4). (**b**) Glucose-stimulation at the indicated time causes the de-SUMOylation of GFP-tomosyn1 and reduced syntaxin1 binding in INS 832/13 cells (representative of n = 3). (**c**) Co-immunoprecipitation of protein lysates from INS 832/13 cells expressing GFP-tomosyn1 demonstrate K298 as a site for SUMO1 modification (representative of n = 3). (**d**) K298R reduces the interaction of GFP-tomosyn1 and TAT-Hisx6-SUMO1 captured by Ni-NTA resin in INS 832/13 cells (representative of n = 4). (**e**) Loss of tomosyn1 SUMOylation in the K298R mutant results in reduced binding to syntaxin1 (representative of n = 3). (**f**) The wild-type GFP-tomosyn1 (WT) and the K854R mutant reduces the glucose-dependent amplification of exocytosis from human β-cells, however the K298R mutant lacks this suppressive effect (n = 35–46, from 4 donors). 1 mM glucose (1G, grey traces/bars) and 10 mM glucose (10G, black traces/bars). **p < 0.01, and ***p < 0.001.

We observed by immunoprecipitation of whole cell INS 832/13 (Fig. 1a) and human islet lysates (Fig. S1a) that SUMO1 is pulled down together with tomosyn (referring to all tomosyn isoforms since the antibody used does not distinguish between isoforms). Furthermore, using a GFP-tagged tomosyn1 (GFP-Tomosyn1) in INS 832/13 cells, this interaction is reduced upon stimulation with 10 mM glucose (Fig. 1b). This occurs in concert with reduced binding of syntaxin1 (Fig. 1b), which contains two putative SUMO-interacting motifs (SIMs; Fig. S1b).

*In-silico* prediction of SUMOylation sites in human, mouse, and rat tomosyn sequences revealed that the consensus PILKVEX motif within tomosyn1/2 is highly conserved among these species (Fig. S1c). The non-consensus PEEKDEK motif is only present in the human and rat tomosyn1 sequence. Thus, we introduced K298R in PILKVEX motif, K854R in PEEKDEK motif, or both mutations into rat GFP-tomosyn1 by site-directed mutagenesis. We observed by immunoprecipitation that the K298R, but not K854R, strongly impaired GFP-tomosyn1 interaction with SUMO1 (Fig. 1c), suggesting that SUMOylation by SUMO1 occurs at K298. To confirm this in a separate assay, INS 832/13 cells were transfected with GFP-tomosyn1 constructs, then incubated with a recombinant TAT-Hisx6-SUMO1. SUMOylated proteins were captured by Ni-NTA resin (Fig. S1d). Again the K298R mutation, but not K854R, decreased the binding of TAT-Hisx6-SUMO1 to GFP-tomosyn1 (Fig. 1d). Finally, SUMOylation of tomosyn1 K298 is required for binding to syntaxin1 (Fig. 1e), but not to SNAP25 (Fig. S1e).

Following a pre-incubation at 1 mM glucose, acute exposure to 10 mM glucose amplifies the exocytotic response of human ß cells to a series of membrane depolarizations^3, 4^. Overexpression of tomosyn1 inhibits this response (Fig. 1f), similar to its ability to block insulin exocytosis in rodents^7, 8^. SUMOylation at K298 is required for this, while the K854 had no effect (Fig. 1f). Taken together, these results show that tomosyn1 is SUMOylated at K298, and this is required for the suppression of ß cell exocytosis.

### Tomosyn-1 interacts with the secretagogin in pancreatic ß cells

Tomosyn is generally thought to sequester syntaxin1A and thus inhibits its binding to SNAP25, a limiting step required for assembly of the minimal SNARE complex^12^. However, the inhibitory role of tomosyn does not only depend on syntaxin binding^20^. Therefore, we sought to identify additional tomosyn1-interacting proteins. GFP-tomosyn1 was expressed in INS 832/13 cells under standard culture conditions, followed by immunoprecipitation coupled to LC MS/MS proteomic analysis. We detected 88 tomosyn1-interacting proteins (Table S1).

**Figure 2:**
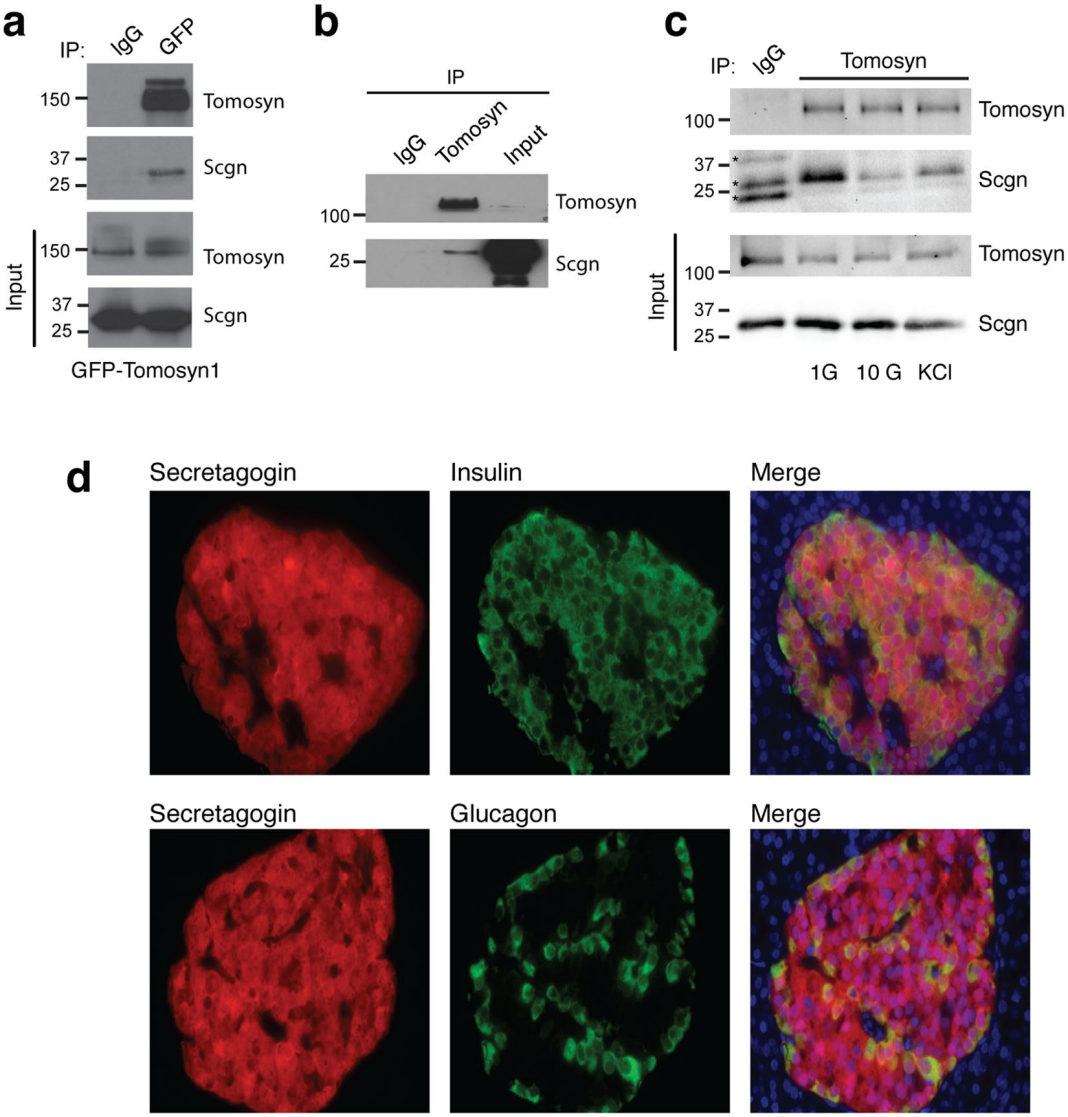
Tomosyn and secretagogin interaction decreases in response to insulin secretagogues. (**a**) Pull-down of GFP-tomosyn1 demonstrates an interaction with secretagogin (Scgn) in INS 832/13 cells (representative of n = 4). (**b**) Pull-down of native tomosyn demonstrates an interaction with secretagogin in human islets (representative of n = 3). (**c**) Stimulation with 10 mM glucose and 30 mM KCl for 15 minutes reduces the tomosyn and secretagogin interaction (representative of n = 3). (**d**) Secretagogin is expressed in ß- and α-cells as shown by immunostaining of human pancreatic sections for secretagogin (red) and insulin (green, *top*) or glucagon (green, *bottom*) (representative of pancreatic section from 6 human donors). *Unspecific bands.

Since tomosyn regulates insulin secretion in a Ca^2+^-dependent manner^9^ we focused on the interaction of tomosyn with secretagogin, a hexa EF-hand Ca^2+^-binding protein^21^ shown to interact with SNARE proteins such as SNAP25 in a Ca^2+^-dependent manner^22^. We confirmed the interaction of GFP-tomosyn1 and secretagogin in INS 832/13 cells (Fig. 2a). Additionally, native secretagogin is pulled down with native tomosyn in isolated human islets (Fig. 2b). Furthermore, the GFP-tomosyn1 and secretagogin interaction in INS 832/13 cells decreased in response to glucose- or KCl-stimulation by 44.6% ± 8.5 and 65.3% ± 13.4, respectively (Fig. 2c). Interaction of syntaxin 1A with tomosyn, however, is not significantly reduced by KCl in INS 832/13 cells (Fig. S2a). This suggests that secretagogin dissociates from tomosyn under conditions that stimulate Ca^2+^-entry and insulin secretion, while the interaction with syntaxin 1A is more glucose-dependent.

Secretagogin was initially cloned from neuroendocrine and pancreatic islet cells^21^, and we confirm that expression of secretagogin in human pancreas is limited to insulin- and glucagon-positive cells (Fig. 2d). Similar secretagogin expression was also observed in mouse islets (Fig. S2b). Although secretagogin is reduced in islets from GK diabetic rat^23^, high-fat fed mice^24^, and isolated islets exposed to high glucose^25^, we find that secretagogin mRNA (Fig. S2c,d; Table S2) or immunostaining (Fig. S2i) between donors with and without type 2 diabetes (T2D) was not different. We also observed no correlation between secretagogin mRNA levels and glycaemia (Fig. S2e,f) or body mass index (Fig. S4g,h). Thus, the expression of secretagogin is not altered in islets from donors with T2D.

### Secretagogin controls insulin secretion by facilitating depolarization-induced exocytosis

**Figure 3:**
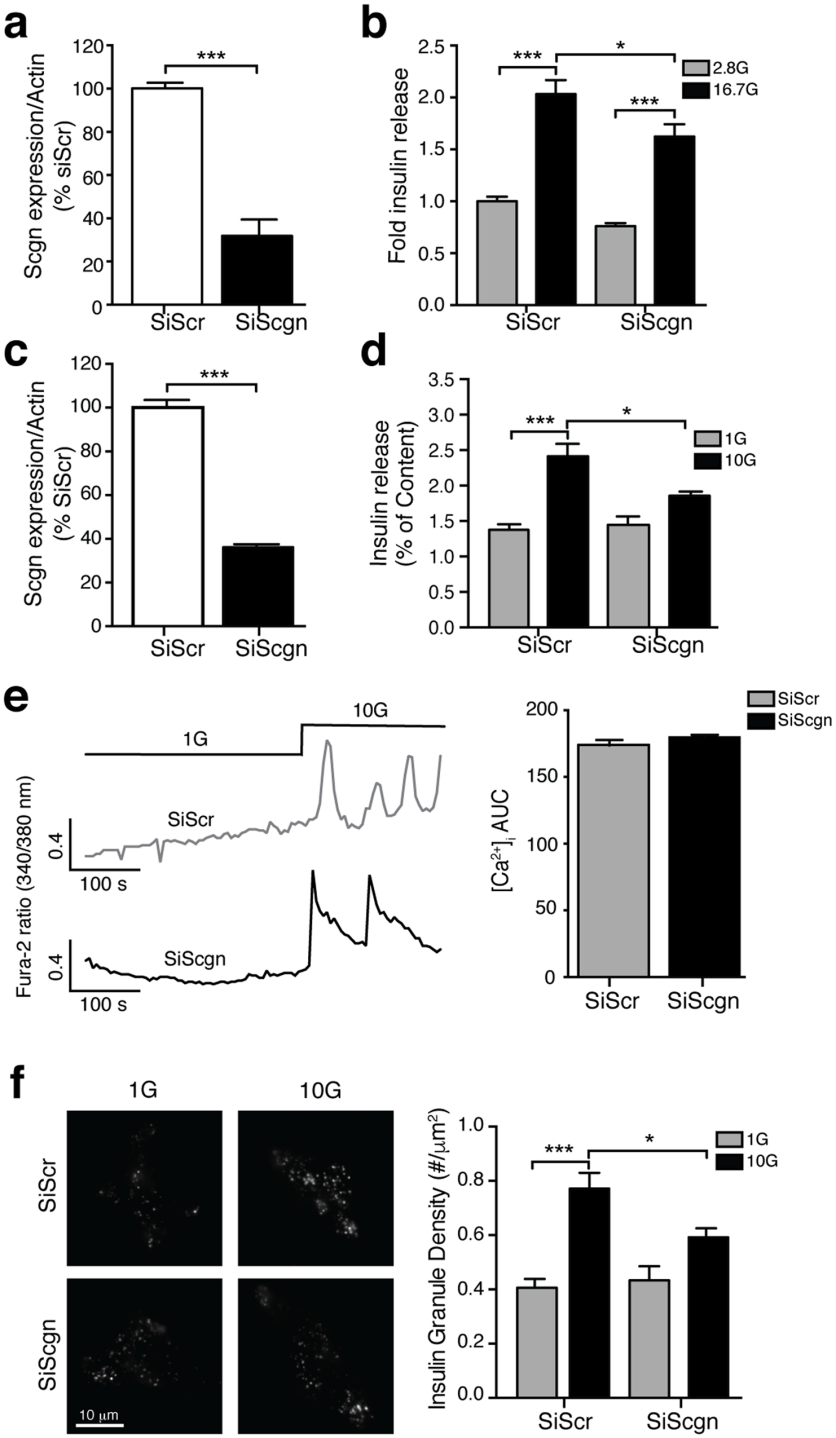
Secretagogin controls insulin secretion downstream Ca^2+^ influx. (**a–d**) Transient transfection of dispersed human islet (**a,b**) and INS 832/13 cells (**c,d**) with SiScgn reduced mRNA expression (**a,c**) and glucose-induced insulin secretion (**b,d**). (**e**) The glucose-induced [Ca^2+^]_i_ response is not modified upon reduction of secretagogin expression (n = 158, 166 cells from 3 independent experiments). (**f**) The recruitment of insulin granules marked by NPY-Venus in response to 10 mM glucose for 15 minutes is reduced in INS 832/13 cells transfected with SiScgn. (n = 32–46 cells from 4 independent experiments). *p < 0.05, ***p < 0.001.

Given that the interaction between secretagogin and tomosyn is regulated by glucose and KCl, we explored the role of secretagogin in insulin secretion. In dissociated human islets and INS 832/13 cells secretagogin mRNA expression was reduced by 50 ± 6% and 63 ± 2%, respectively, by siRNA directed against secretagogin (SiScgn) compared with a scrambled control (SiScr; Fig. 3a,c). Insulin content (Fig. S2j) and basal insulin secretion at 1 mM glucose (Fig. 3b,d) were unchanged. However, knockdown of secretagogin reduced glucose-induced insulin secretion from dissociated human islets and INS 832/13 cells by 20 ± 7% and 23 ± 2%, respectively (Fig. 3b,d).

**Figure 4:**
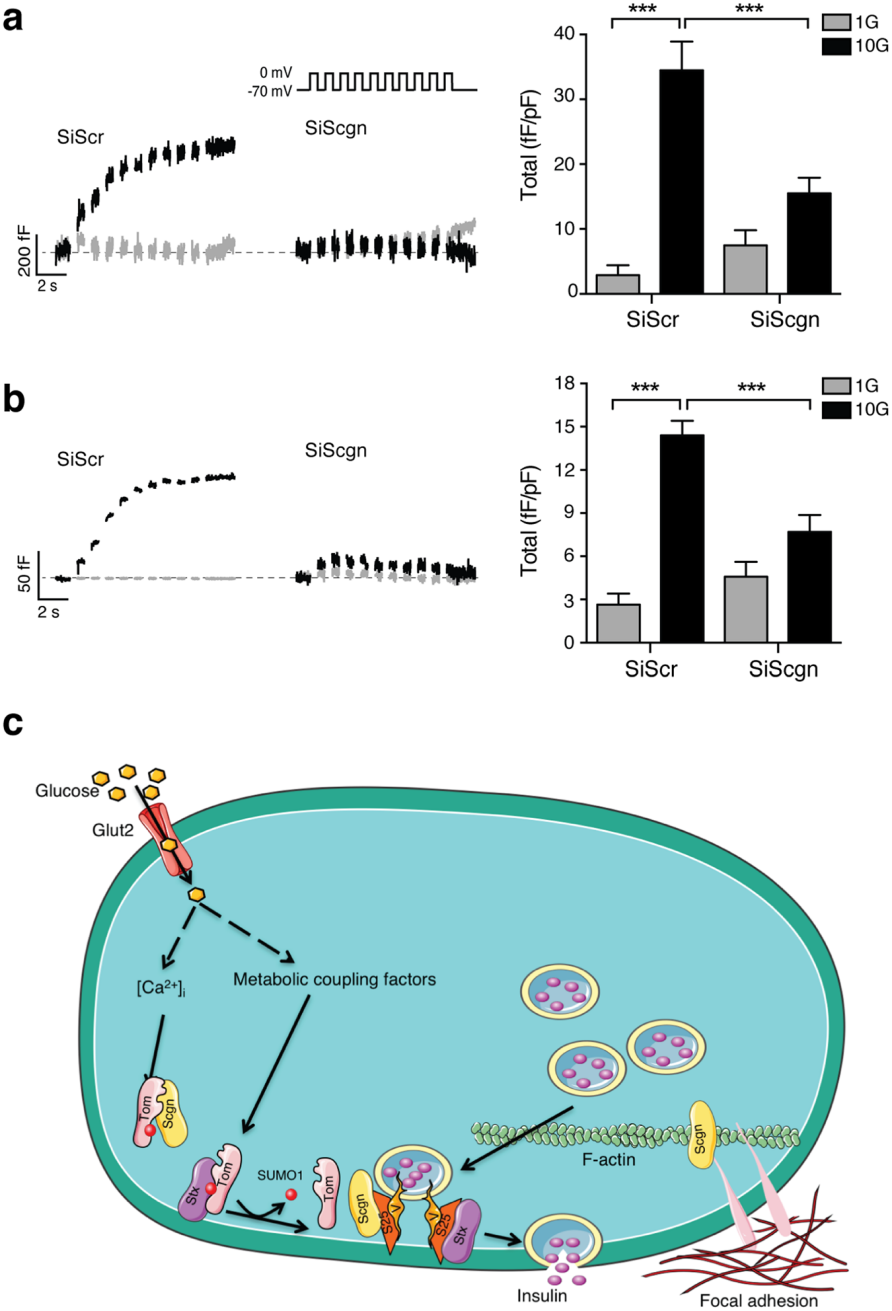
Secretagogin controls insulin secretion downstream of Ca^2+^ influx. Transient transfection of SiScgn reduced the glucose-dependent amplification of exocytosis from human β-cells (**a**); n = 19–41 cells, from 3 donors) and INS 832/13 cells (**b**), n = 35–46 cells, from 3 experiments). 1 mM glucose (1G, grey traces/bars) and 10 mM glucose (10 G, black traces/bars). ***p < 0.001. (**c**) Schematic illustration of the mechanism by which tomosyn and secretagogin regulate insulin secretion process. Glut2: Glucose transporter. Scgn: secretagogin, Tom: Tomosyn, S25: SNAP25, V: VAMP2, SENP1: Sentrin-specific protease 1.

Knockdown of secretagogin does not affect the basal or glucose-stimulated increase of [Ca^2+^]_i_ in INS 832/13 cells (Fig. 3e), suggesting an action downstream of Ca^2+^-entry. Next we measured the density of secretory granules, visualized by expression of NPY-Venus, at the plasma membrane by total internal reflected fluorescence (TIRF) microscopy in INS 832/13 cells. The density of insulin granules at 1 mM glucose was not affected by the reduction of secretagogin (Fig. 3f). However, glucose-induced insulin granule recruitment was impaired by 23 ± 1% in SiScgn transfected cells (Fig. 3f), showing that secretagogin is important for insulin granule recruitment. Accordingly, knockdown of secretagogin impaired the ability of glucose to facilitate depolarization-induced exocytosis. As exocytosis at 10 mM glucose was reduced by 55 ± 1% and 46 ± 7% in human ß cells (Fig. 4a) and INS 832/13 cells (Fig. 4b), respectively, while exocytosis elicited at 1 mM glucose was unaffected.

## Discussion

Tomosyn is a negative regulator of exocytosis and secretion in insulin-secreting cell lines^7^, and mouse ß cells^8^. Here we show that the post-translational modification of tomosyn by SUMO1 is required for syntaxin1A binding and inhibition of insulin exocytosis. In parallel the Ca^2+^-binding protein secretagogin is required for insulin granule recruitment and exocytosis, and interacts with tomosyn in a Ca^2+^-dependent manner. This places tomosyn as an important integrator of metabolic coupling signals, via the mitochondrial export of reducing equivalents and subsequent control of SUMOylation, and Ca^2+^-raising signals.

Post-translational modifications play a key role regulating assembly of the exocytotic machinery^13^ and SUMO1 in particular controls insulin exocytosis in response to metabolic signals, likely by regulating events at the plasma membrane^4^. Consistent with these observations, we showed that tomosyn is modified by SUMO1, is de-SUMOylated upon glucose-stimulation, and this controls its interaction with syntaxin1. Furthermore, loss of SUMOylation at K298 in the PILKVEX consensus motif in rat tomosyn1 reduces SUMO1-conjugation in concert with syntaxin1 binding, which is accompanied with a de-repression of exocytosis from human ß cells. Therefore, the de-SUMOylation of tomosyn alleviates its inhibitory function, possibly by favoring the displacement of syntaxin1A to interact with SNAP25, a key step required for the formation of the minimal exocytotic complex^5^. Another proposed model has suggested that tomosyn inhibits exocytosis through the formation of tomosyn-SNAP25 complexes^15–31^. However, the K298 does not displace SNAP25 from tomosyn-1 (Fig. S1e), likely because of the absence of a SUMO-interacting motif in SNAP25 and because the SUMOylation site on tomosyn is far from the SNPA25-binding domains at amino acids 537–578 and 897–917.

Although syntaxin1A is a well-known binding partner of tomosyn, it is clear that tomosyn mediates its inhibitory effect in part independently of binding syntaxin1A^20^ and in a Ca^2+^-dependent manner^9^. We therefore identified multiple tomosyn-interacting proteins, including the Ca^2+^-binding protein secretagogin which was also suggested to interact with tomosyn2 in previous proteomic data along with several other overlapping proteins identified in our dataset (indicated in Table S1)^13^, and potential novel interactors including several of the 14-3-3 scaffold proteins (although these interactions remain to be confirmed). Secretagogin is an EF-hand Ca^2+^-binding protein initially identified in neuronal and islets cells^21^ that we show to be restricted to α- and ß-cells in the pancreas. In contrast to previous observations in islets from diabetic GK rats^23^, high fat fed mice^24^, and in islets exposed to chronic high glucose^25^, we did not observe any alteration of secretagogin levels, either mRNA or protein, in islets from human T2D donors. Given that exocytosis can be rescued in T2D ß cells by re-introduction of metabolic coupling factors^3^, it is tempting to suggest that the exocytotic machinery itself is not limiting in T2D, but rather the upstream regulatory mechanisms represent the main dysfunction in T2D.

Knockdown of secretagogin reduces exocytosis and insulin secretion from human ß cells and INS 832/13 cells. This is consistent with recent studies showing that the secretagogin controls insulin secretion from MIN6^26^ and NIT1 cells^27^. In agreement with the observation that secretagogin controls F-actin filament remodeling, glucose-induced focal-adhesion, and TAU protein that controls microtubule dynamics^27, 28^, we find that secretagogin is required for the glucose-dependent recruitment of insulin granules to the plasma membrane.

Secretagogin is a Ca^2+^ sensing protein that changes its conformation when [Ca^2+^]_i_ rises, allowing its interaction with exocytotic proteins like SNAP25 and Doc2alpha^29^. Accordingly, we observed that secretagogin interacts with tomosyn under basal conditions, and this is decreased in response to glucose and KCl, the latter finding suggesting an important role for [Ca^2+^]_i_ as opposed to glucose-dependent metabolic signals. We find the absence of a putative SIM domain on secretagogin (Fig. S2k), suggesting that secretagogin is more likely not binding to the SUMOylated motif of tomosyn (Fig. 4c). We suggest that tomosyn sequesters secretagogin and syntaxin1A under basal conditions as a ‘brake’ on insulin exocytosis. We thus propose (Fig. 4c) that in response to insulin secretagogues, the metabolism-driven de-SUMOylation by SENP1 and increase in Ca^2+^ promote the release of syntaxin1A and secretagogin and formation of an exocytotic complex, the regulation of the F-actin remodeling, and focal adhesion complexes to control glucose-dependent insulin secretion.

## Methods

### Human islet isolation and INS 832/13 cells culture

Human islets were isolated from donor organs at the Alberta Diabetes Institute IsletCore (www.bcell.org/IsletCore.html) or the Clinical Islet Laboratory at the University of Alberta as described^30^ and cultured as described^3^. Diabetes status was determined by patient clinical history and by HbA1c measurement (we determine undiagnosed T2D if HbA1c is higher that 7.0%). A detailed list of donors is provided in Table S2. The collection of, and experiments on, human tissue were approved by the Human Research Ethics Board (Pro00001754) at the University of Alberta, and all experiments were carried out in accordance with relevant guidelines and regulations at the University of Alberta. Donors or their relatives provided written informed consent to the use of pancreas tissue for research. INS 832/13 cells, a kind gift from Dr. Chris Newgard (Duke University), were cultured as described^3^.

### siRNA and plasmid transfection

Human pancreatic islets were dissociated using Cell Dissociation Buffer enzyme-free, Hanks’ Balanced Salt Solution (ThermoFisher Scientific. Burlington, ON). Dissociated human islets cells and INS 832/13 cells were transfected with scramble siRNA (SiScr) and siRNA against secretagogin (SiScgn) using DharmaFECT 1 (GE Healthcare) according to manufacturer’s protocol (see Supplemental Materials for additional details). In patch-clamp studies, a fluorescent siRNA (Cat # 1027284, Qiagen, Toronto, Canada) was included to identify transfected cells. INS 832/13 cells or dispersed human islet cells were transfected with GFP or GFP-tomosyn1 plasmids (generously given by Dr. Romano Regazzi, University of Lausanne, Switzerland) using Lipofectamine 2000.

### Co-immunoprecipitation, western blotting, and LC MS/MS analysis

Human islets or INS 832/13 cells were incubated in regular media or treated with glucose or KCl in KRB solution containing (in mM) 115 NaCl, 5 KCl, 24 NaHCO_3_, 2.5 CaCl_2_, 1 MgCl_2_, 10 HEPES, and 0.1% BSA, PH 7.4) as indicated. Then, cells were harvested in lysis buffer containing (in mM) 20 Tris-HCl (pH = 7.5); 150 NaCl, 1 EDTA, 1 EGTA, 2.5 sodium pyrophosphate, 1 EDTA, 1 b-glycero-phosphate, 25 N-ethylmaleimide, 1% Triton X-100, and 1X protease inhibitor cocktail. 300 µg of cell extract were incubated overnight with rotation with 0.25 µg of tomosyn or 1 µl of crude serum GFP antiserum, and 30 μl of protein G agarose in PBS 1X (1:1 slurry). Normal mouse and goat serum was used as negative control. Beads were then spun at 2000 rpm and washed 4 times with PBS 1X. Immunprecipitated proteins were eluted in 3X laemmeli buffer. 1/6 of immunoprecipitated protein and 30 μg of input proteins were separated using SDS-PAGE, transferred to nitrocellulose membrane, and probed with indicated antibodies.

### Insulin secretion

Human islets were dissociated using Accutase (Gibco) and plated at a density of 5000 cells/well in a 96 Vwell plate and transfected with siRNA at the time of plating. INS 832/13 cells were seeded at 70% confluence in 24 wells plates and transfected the day after. Two days after transfection, cells were starved with 1mM KRB solution for 45 min for human cells, and 1 hour for INS 832/13 cells, followed by 1hr stimulation with either 1 or 16.7 mM glucose KRB. Insulin content was extracted with acid-ethanol solution (1.5% concentrated HCl, 23.5% acetic acid, and 75% ethanol) overnight at 20 °C. Samples were assayed using the Insulin Detection Kit (Meso Scale Discovery. Rockville, MD).

### Exocytosis

Three days after transfection, cells were pre-incubated in 1 mM glucose regular media for an hour. Then, media was changed to a bath solution that contained (in mM): 118 NaCl, 20 TEA, 5.6 KCl, 1.2 MgCl_2_•6H_2_O, 2.6 CaCl_2_, 5 HEPES, and either 1 or 10 glucose (pH 7.4 with NaOH) prior to patch-clamping in a heated chamber (32–35 °C). Single green cells were used for whole-cell patch-clamp measurement of exocytosis as described previously^3^. The intracellular solution contained (in mM): 125 Cs-glutamate, 10 CsCl, 10 NaCl, 1 MgCl_2_•6H_2_O, 0.05 EGTA, 5 HEPES, 0.1 cAMP, and 3 MgATP (pH 7.15 with CsOH).

### Imaging

For [Ca^2+^]_i_ imaging, INS 832/13 cells were incubated for 1 hour with 3 μM Fura 2-AM and 0.06% pluronic acid (Life Technologies) in solution containing (in mM) 130 NaCl, 5 KCl, 2 CaCl_2_, 1 MgCl_2_, 5 NaHCO_3_, 10 HEPES, 1 glucose (pH 7.4). Cells were imaged in 1 mM glucose at 37 °C with constant bath perfusion. Glucose was increased to 10 mM, as indicated. Imaging was performed with a Stallion imaging system (Olympus Canada, Richmond Hill, ON, Canada) and Ratio Cam software (Metamorph; Molecular Devices, Sunnyvale, CA). Dual excitation at 340 and 380 nm was used, and emission at 510 nm measured.

For TIRF microscopy the SiScr or SiScgn, and NPY-Venus construct, were co-transfected in INS 832/13 cells using Lipofectamine 2000. Cells were plated on coverslips, treated in KRB solution as indicated, fixed with Z-fix after 48 hours, and visualized using a Cell-TIRF motorized TIRF system (Olympus Canada) with a 100×/1.49 NA TIRFM objective, a Photometrics Evolve 512 camera (Photometrics), and Metamorph Imaging software (Molecular Devices). Stimulation was at 491 nm and the penetration depth was set to 110 nm, calculated using existing angle of the laser from the objective and assuming a refractive index of 1.37. Granule density was analyzed with Image-Pro Plus software (Rockville, MD, USA). Immunofluorescence of human and mouse pancreas sections were performed and quantified as previously described^3^.

### Statistical analysis

Data analysis was performed using GraphPad Prism (v6.0c). Comparison of multiple groups was by one-or two-way ANOVA followed by Bonferroni post-test. When comparing two means only, data were analyzed by the 2-tailed Student’s t test. A P value less than 0.05 was considered significant.

## Electronic supplementary material

Supplementary Information

Supplementary Table 1
