# Supplementary material for: SUMOylation and calcium control syntaxin-1A and secretagogin sequestration by tomosyn to regulate insulin exocytosis in human ß cells
Source: Sci Rep. 2017 Mar 21;7:248. doi: 10.1038/s41598-017-00344-z (PMC5428262; doi:10.1038/s41598-017-00344-z)
Supplement: Supplementary file 1 — Supplementary Information [file 41598_2017_344_MOESM1_ESM.pdf]

Supplementary Information for:

SUMOylation and calcium control syntaxin-1A and secretagogin sequestration by tomosyn to regulate insulin exocytosis in human ß cells.

Mourad Ferdaoussi*, Jianyang Fu, Xiaoqing Dai, Jocelyn E. Manning Fox, Kunimasa Suzuki, Nancy Smith, Gregory Plummer, Patrick E. MacDonald*.

Alberta Diabetes Institute and Department of Pharmacology, University of Alberta, Edmonton, Alberta, Canada, T6G 2R3.

Supplemental Experimental Procedures.

Reagents. All chemical reagents were from Sigma Aldrich (Oakville, ON, Canada), expect NaCl, HEPES and $NaHCO_3$ were from Fisher Scientific (Ottawa, Ontario, Canada), and EGTA and EDTA from EMD Millipore (Etobicoke, ON, Canada). Scramble siRNA (SiScr; #4390846) and siRNA directed against Human and rat secretagogin (SiScgn; s20791 and s157840, respectively) were from ThermoFisher Scientific (Rockford, IL, USA). To identify transfected cells in patch-clamp experiments, cells were co-transfected with a fluorescent siRNA (Cat# 1027284,, Qiagen, Toronto, Canada). Goat GFP antiserum was form Eusera (cat #EU3). Mouse anti-tomosyn was from BD Biosciences (cat #611296, Mississauga, ON, Canada). Mouse anti-Syntaxin1 (cat #sc-12736), anti-SUMO1 (cat #sc-5308), and normal mouse (cat #sc-2025) and goat IgG (cat #sc-2028) were from Santa Cruz biotechnology (Dallas, TX, USA). Rabbit anti-secretagogin was form ABCAM (cat #ab137017, Toronto, ON, Canada). Protein G was from GE Healthcare Life Sciences (cat # 17-0618-01, Mississauga, ON, Canada). His-tag antibody (cat # MA1-21315), Fura 2-AM (cat # F1221) and Tris were from Thermo Fisher Scientific.

LC MS/MS analysis. INS 832/13 cells cultured in regular media with 11.1 mM glucose were harvested and tomosyn was pulled down as indicated. Immunprecipitated proteins were eluted in 3X laemmeli buffer and separated using SDS-PAGE. Gels were stained with coomassie blue, and 5 prominent bands located between 75 and 25 KDa were

excised and sequenced separately by the Alberta Proteomics and Mass Spectrometry Facility.

Quantitative PCR (qPCR). RNA was isolated from human islets and INS 832/13 cells using Trizol according to manufacturer's protocol. cDNA was synthetized using the 5X All-In-One RT MasterMix (ABM, Richmond, BC) and qPCR was performed using the SYBR master mix (ThermoFisher Scientific) using the following primers. Human Scgn: forward, 5'-CACCATGGATTCTGGCTCTT-3', and reverse, 5'-CTTCCAGGGC TCCTGTTTTA-3'; human β-actin: forward, 5'-GGACTTCGAGCAAGAGATGG-3', and reverse, 5'-AGCACTGTGTTGGCGTACAG-3'; human TBP: forward, 5'-GAACCACGG CACTGATTTTC-3', and reverse, 5'-GCTGGAAAACCCAACTTCTG-3'; rat Scgn: forward, 5'-TTGTTCTTTCGCCTGGAAAC-3', and reverse, 5'-TGGTGGAGGAAAAG ATCTCG-3; rat actin: forward, 5'-TGAAGTGTGACGTTGACATCC -3 and reverse, 5'-ACAGTGAGGCCAGGATAGAGC-3.

TAT-Hisx6-SUMO1 cloning, production, purification. The pTAT-T7-SUMO1 expression vector, which is a parental plasmid of pTAT-Hisx6-SUMO1, was prepared by inserting SUMO1 cDNA between BamH-I and Not-I of pTAT-CRE (gift from Steven Dowdy, Addgene plasmid # 35619). Double stranded oligonucleotide encoding Hisx6 tag was inserted between Nde-I and BamH-I of pTAT-T7-SUMO1. The recombinant TAT-Hisx6-SUMO1 protein was expressed in Rosetta (DE3)pLysS (Novagen-EMD Millipore, MA, USA) using 0.5 mM IPTG for the induction. The E.coli pellet-containing TAT-Hisx6-SUMO1 was homogenized in 6 M guanidine HCl, 0.1 M sodium acetate, pH 4

using a ULTRA-TURRAX homogenizer (IKA Inc., NC, USA). Total protein was precipitated by addition of 3 volumes of water, 40% methanol and 10% chloroform. Protein pellet was dissolved in 6 M guanidine HCl, 25 mM Tris, 0.5% Triton X-100, pH 8 and then loaded onto a nickel-NTA column (1 x 3 cm, QIAGEN, Hilden, GmbH). After washing the column with sample buffer, TAT-Hisx6-SUMO1 was eluted with 6 M guanidine HCl, 0.1 M imidazole, pH 8. Recombinant TAT-Hisx6-SUMO1 was reduced with 50 mM of DTT at room temperature overnight, and then dialyzed against PBS. INS 832/13 cells were treated with 1 µM of TAT-Hisx6-SUMO1 peptide for 6 and 24 hours for Fig S1d, and 16 hours for Fig 1d.

References:

Zhao, Q., Xie, Y., Zheng, Y., Jiang, S., Liu, W., Mu, W., Liu, Z., Zhao, Y., Xue, Y., Ren, J., 2014. GPS-SUMO: a tool for the prediction of sumoylation sites and SUMO-interaction motifs. Nucleic Acids Res. 42, W325–30.

Supplemental Figure Legends.

Figure S1 related to Figure 1:

a: Pull-down of native tomosyn demonstrates the co-immunoprecipitation of SUMO1 in human islets (representative of n=4).

b: Domain diagram of Syntaxin1A showed the putative SUMO-interacting domains (SIM), α-helices domains (HA, HB, and HC), SNARE motif (SNARE, and the transmembrane domain (TMR).

c: *In silico* analysis of the putative SUMOylation site (yellow boxes) of human and rodent tomosyn1 (STXB5) and tomosyn2 (STB5L) using the GPS-SUMO prediction tool (http://sumosp.biocuckoo.org/online.php, (Zhao et al., 2014)).

d: The SUMOylation of the cytoplasmic and nuclear target proteins of INS 832/13 cells increases after treatment with recombinant TAT-Hisx6-SUMO1.

e: Co-immunoprecipitation of protein lysates from INS 832/13 cells expressing GFP-tomosyn1. The interaction of SNAP25 was not altered by the K298R and K847R mutations (representative of n=4).

Figure S2 related to Figure 2:

a: The stimulation of INS 832/13 cells with 30 mM KCl for 15 minutes reduces the tomosyn and syntaxin 1 interaction (representative of n=3).

b: Secretagogin is expressed in ß- and α-cells as shown by immunostaining of mouse pancreatic sections for secretagogin (red) and insulin (green, *top*) or glucagon (green, *bottom*) (representative of pancreatic sections from 3 mice).

c and d: The secretagogin mRNA levels normalized to TBP **(c)** or ß-actin **(d)** are similar in islets from T2D and non-diabetic donors. **e-h:** Correlation of secretagogin mRNA levels normalized to TBP **(e and g)** or ß-actin **(f and h)** with HbA1c **(e and f)** or BMI **(f and h**). **I:** Secretagogin protein levels measured by immunohistochemistry were similar in islets from T2D and non-diabetic donors. Values are mean ± SEM (n =3-9).

j: Insulin content was not altered in INS 832/13 cells transiently transfected with SiScgn (n=3).

k: Domain diagram of secretagogin showing the calcium binding domains (CB), and the absence of SIM domain.

Table S1 related to Figure 1: Tomosyn1-interacting protein identified by immunoprecipitation coupled to MS-MS LC proteomic sequencing. See attached Excel file.

Table S2 related to Figures 1-4: Human islet donor information.

Table S2 related to Figures 1-4: Human islet donor information. Donors beginning with "R" were processed by the Alberta Diabetes IsletCore, and donors beginning with "H" were processed by the Clinical Islet Laboratory at the University of Alberta.

Donor ID	Age (years)	Sex	BMI	HbA1c (if known)	T2D, y/n (years, if known)	T2D medication (if known)
R022	48	F	18.0		N	
R023	65	M	29.3	8.3	Y	
R024	52	F	29.0		Y	
R030	80	F	21.9		N	
R031	54	M	30.0	7.2	Y	
R045	27	F	19.5	5.2	N	
R054	73	F	22.4	6.1	Y (5 yrs)	
R057	53	F	35.5	10.3	Y (20 yrs)	Metformin
R063	57	F	23.0	6.1	Y (2 yrs)	
R064	36	M	28.1	10.9	Y (1.5 yrs)	
R086	45	M	23.8	7.4	Y (2.5 yrs)	
R088	20	M	40.9	5.8	N	
R107	56	F	27.5	9.3	Y	
R109	60	F	25.0	6.1	N	
R110	41	M	26.8	9.3	Y (2 yrs)	Metformin
H1557	27	F	21.6	5.8	N	
H1621	27	M	35.0	5.8	N	
H1687	21	M	23.2		N	

Figure S1

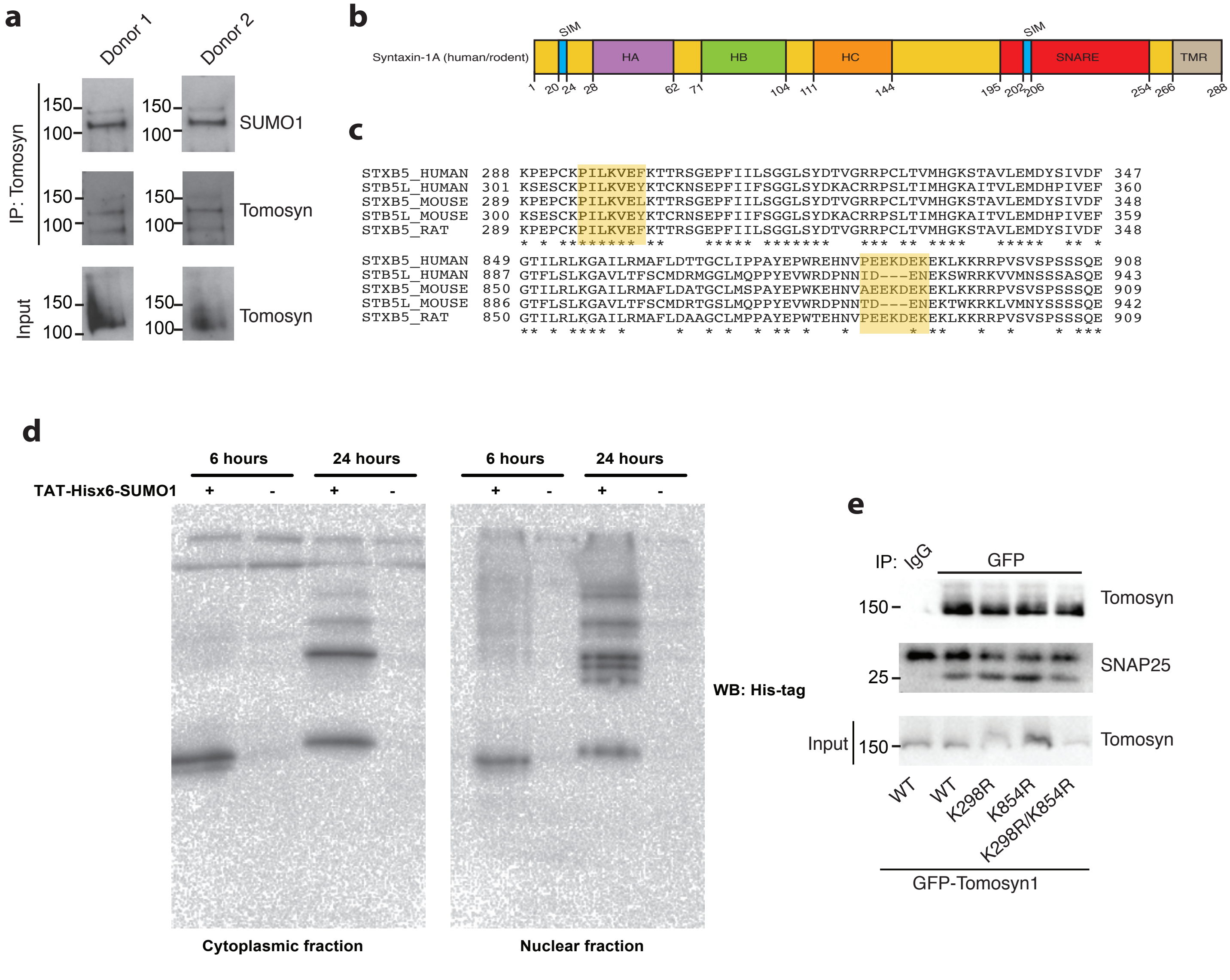
a
Donor 1
Donor 2
IP: Tomosyn
150
100
SUMO1
Tomosyn
Input
b
Syntaxin-1A (human/rodent)
SIM
HA
HB
HC
SNARE
TMR
1 20 24 28 62 71 104 111 144 195 202 206 254 266 288
c
STXB5_HUMAN 288 KPEPCKPILKVEFKTTRSGEPFIILSGGLSYDTVGRRPCLTVMHGKSTAVLEMDYSIVDF 347
STB5L_HUMAN 301 KSESCKPILKVEYKTCKNSEPFIIFSGGLSYDKACRRPSLTIMHGKAITVLEMDHPIVEF 360
STXB5_MOUSE 289 KPEPCKPILKVELKTTRSGEPFIILSGGLSYDTVGRRPCLTVMHGKSTAVLEMDYSIVDF 348
STB5L_MOUSE 300 KSESCKPILKVEYKTCRNSEPFIIFSGGLSYDKACRRPSLTIMHGKAITVLEMDHPIVEF 359
STXB5_RAT 289 KPEPCKPILKVEFKTTRSGEPFIILSGGLSYDTVGRRPCLTVMHGKSTAVLEMDYSIVDF 348
STXB5_HUMAN 849 GTILRLKGAILRMAFLDTTGCLIPPAYEPWREHNVPEEKDEKEKLKKRRPVSVSPSSSQE 908
STB5L_HUMAN 887 GTFLSLKGAVLTFSCMDRMGGLMQPPYEVWRDPNNID---ENEKSWRRKVVMNSSSASQE 943
STXB5_MOUSE 850 GTILRLKGAILRMAFLDATGCLMSPAYEPWKEHNVAEEKDEKEKLKKRRPVSVSPSSSQE 909
STB5L_MOUSE 886 GTFLSLKGAVLTFSCMDRTGSLMQPPYEVWRDPNNTD---ENEKTWKRKLVMNYSSSSQE 942
STXB5_RAT 850 GTILRLKGAILRMAFLDAAGCLMPPAYEPWTEHNVPEEKDEKEKLKKRRPVSVSPSSSQE 909
d
6 hours
24 hours
TAT-Hisx6-SUMO1
+
-
WB: His-tag
Cytoplasmic fraction
Nuclear fraction
e
IP: IgG
GFP
150
25
Tomosyn
SNAP25
Input
WT
WT
K298R
K854R
K298R/K854R
GFP-Tomosyn1

Figure S2

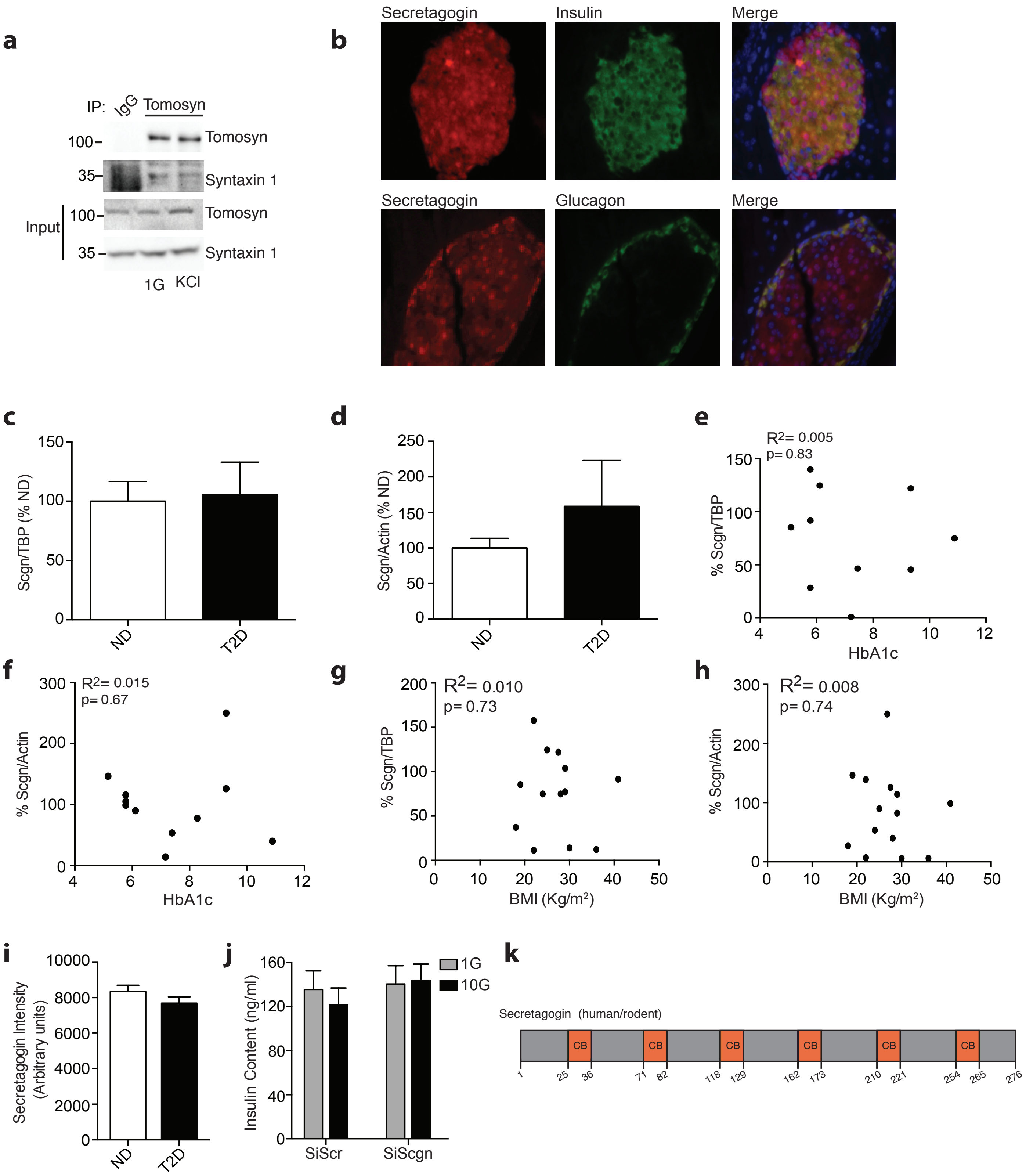

a
IP: IgG Tomosyn
100
35
Input
Tomosyn
Syntaxin 1
1G KCl
b
Secretagogin
Insulin
Merge
Glucagon
c
Scgn/TBP (% ND)
ND
T2D
d
Scgn/Actin (% ND)
e
R^2= 0.005
p= 0.83
% Scgn/TBP
HbA1c
f
R^2= 0.015
p= 0.67
% Scgn/Actin
g
R^2= 0.010
p= 0.73
BMI (Kg/m^2)
h
R^2= 0.008
p= 0.74
i
Secretagogin Intensity (Arbitrary units)
j
Insulin Content (ng/ml)
1G
10G
SiScr
SiScgn
k
Secretagogin (human/rodent)
CB
